# Food-Related Brain Activation Measured by fMRI in Adults with Prader–Willi Syndrome

**DOI:** 10.3390/jcm10215133

**Published:** 2021-10-31

**Authors:** Ingrid Caroline van Nieuwpoort, Tessa N. A. Slagboom, Sigridur Jakobsdóttir, Jan Berend Deijen, Dick J. Veltman, Leopold M. G. Curfs, Madeleine L. Drent

**Affiliations:** 1 Section of Endocrinology, Department of Internal Medicine, Amsterdam UMC, Vrije Universiteit Amsterdam and Amsterdam Neuroscience, De Boelelaan 1117, 1081 HV Amsterdam, The Netherlands; t.slagboom@amsterdamumc.nl (T.N.A.S.); S.Jakobsdottir@zzv.nl (S.J.); ml.drent@amsterdamumc.nl (M.L.D.); 2 Section of Clinical Neuropsychology, Department of Clinical, Neuro- & Developmental Psychology, Faculty of Behavioral and Movement Sciences, Vrije Universiteit Amsterdam, De Boelelaan 1105, 1081 HV Amsterdam, The Netherlands; j.b.deijen@vu.nl; 3 Hersencentrum Mental Health Institute, Marnixstraat 364, 1016 XW Amsterdam, The Netherlands; 4 Department of Psychiatry, Amsterdam UMC, Vrije Universiteit Amsterdam, De Boelelaan 1117, 1081 HV Amsterdam, The Netherlands; dj.veltman@amsterdamumc.nl; 5 Department of Clinical Genetics, Maastricht University Medical Center, Minderbroedersberg 4-6, 6211 LK Maastricht, The Netherlands; leopold.curfs@maastrichtuniversity.nl

**Keywords:** PWS, fMRI, hunger, satiety, leptin, IGF-1, insula, hyperphagia

## Abstract

(1) Background: Prader–Willi syndrome (PWS) is characterized by hyperphagia, resulting in morbid obesity if not controlled. The primary aim of this study was to investigate whether PWS patients show altered activation of brain areas involved in hunger. As a secondary objective, we assessed whether there is an association between these brain areas and several endocrine and metabolic factors in the fasting state. (2) Methods: 12 PWS adults and 14 healthy controls (siblings) performed a food-related experimental task after an overnight fast while brain activation in regions of interest was measured by functional MRI. (3) Results: In controls, significantly more activation was found in the left insula (*p* = 0.004) and the bilateral fusiform gyrus (*p* = 0.003 and 0.013) when the individuals were watching food as compared to non-food pictures, which was absent in PWS patients. Moreover, in PWS adults watching food versus non-food pictures a significant negative correlation for glucose and right amygdala activation (p_fwe = 0.007) as well as a positive correlation for leptin and right anterior hippocampus/amygdala activation (p_fwe = 0.028) was demonstrated. No significant associations for the other hormonal and metabolic factors were found. (4) Conclusions: PWS individuals show aberrant food-related brain activation in the fasting state. Leptin is associated with activation within the neural motivation/reward circuitry, while the opposite is true for glucose.

## 1. Introduction

Prader–Willi syndrome (PWS), caused by abnormalities on chromosome 15, is the most common genetic cause of (life-threatening) obesity, with an estimated prevalence of 1:10,000 up to 1:30,000 individuals [1,2]. PWS is manifested by clinical symptoms such as characteristic facial features, neonatal and infantile hypotonia, short stature with small hands/feet, behavioral abnormalities and mental retardation, high pain threshold and alterations in temperature regulation, and additionally, hypothalamic dysregulation resulting in endocrine deficits [1,2,3,4]. Moreover, these patients experience feeding difficulties and failure to thrive in early childhood, which later on changes to obsession with food and hyperphagia [1,2,3,4]. Most PWS patients become (morbidly) obese when hyperphagia is not controlled at an early stage. Furthermore, when compared to subjects with simple obesity and a comparable body mass index (BMI), the body composition (BC) of obese as well as non-obese PWS patients is found to be aberrant, with decreased muscle and lean body mass and an increased fat mass [5,6,7,8]. Lower total and resting energy expenditure, as a result of decreased lean body mass, may also be a contributing factor in the development of obesity in PWS [9].

Since overweight and obesity are highly associated with a decreased quality of life, morbidity, and eventually even mortality, understanding the factors contributing to the pathogenesis of these conditions is of great importance. Given that PWS patients show profound abnormal eating behavior, knowledge about the regulation of appetite and satiety in this patient group is crucial. However, just like the regulation of food intake and energy balance in general, the exact mechanisms underlying hyperphagia and obesity in PWS are complicated and predominantly unknown. They most likely result from a combination of factors.

First, the central nervous system (CNS) seems to play an important role. Previous positron emission tomography (PET) and functional magnetic resonance imaging (fMRI) studies show that brain areas such as the hypothalamus, thalamus, insula, amygdala, orbital frontal complex (OFC), dorsolateral prefrontal cortex (DLPFC), cingulate, parahippocampal gyrus, and striatum are involved in the processing of food cues and/or the regulation of appetite, satiety, and reward processing [10,11,12,13,14,15,16,17,18]. PWS individuals appear to have aberrant activation of these brain areas. One of the first studies that used a temporal clustering analysis of fMRI in three PWS patients showed a significant delay in the activation of brain areas involved in satiety (i.e., hypothalamus, insula, ventromedial prefrontal cortex (PFC), DLPFC, and nucleus accumbens) after administration of glucose when compared to obese controls [19]. Thereafter, a number of fMRI studies were performed on eating behavior and responses to visual food stimuli in PWS patients, indicating that distinct neural mechanisms could indeed be major factors contributing to the hyperphagia seen in PWS [20,21,22,23,24,25,26,27].

Secondly, the endocrine system is thought to play a key role, with altered levels or actions of metabolic and (an)orexigenic hormonal factors found in PWS [28,29,30,31,32,33]. Since several metabolic and hormonal factors such as triglycerides, glucose, insulin, leptin, and ghrelin relay information on adipose tissue, hunger, and satiety to the previously mentioned brain areas, these factors are also involved in the central regulation of appetite and food intake [11,12,34,35,36,37,38]. Some of the previous research on these factors, eating behavior, and responses to visual food stimuli in PWS patients also confirm distinct neural mechanisms and alterations in hormonal levels/actions, which are major factors contributing to the hyperphagia and obesity seen in PWS [20,21,22,23,24,25,26,27,28,33]. Another common alteration in the endocrine system found in PWS is hypothalamic dysfunction, which leads to endocrine disorders such as hypogonadism and growth hormone deficiency (GHD) [1,2].

The observed alterations in body composition in PWS are similar to those seen in patients with GHD [8]. Indeed, GHD has been described in PWS patients, with varying prevalence. The prevalence of severe GHD is reported to be between 10–50% in the adult PWS population, depending on the diagnostic criteria being used [1,6,7,39,40]. Both growth hormone (GH) and insulin-like growth factor 1 (IGF-1) receptors are widely expressed within the CNS, while the GH/IGF-1 axis is known to be involved not only in brain structure and function, but also in metabolic regulation [41,42,43].

In conclusion, there seems to be a complex interaction between the central nervous system and both (neuro)endocrine and metabolic factors in PWS, which possibly leads to reduced satiety and increased hunger, and thereby the stimulation of hyperphagia and the development of obesity.

To date, little research has been performed on PWS patients, and only small sample sizes, the use of dissimilar control groups, and contradictory results have been reported; therefore, previous findings are difficult to generalize. Furthermore, many studies have focused mainly on satiety and post-meal reward systems. Given that the majority of PWS patients show obsession with food and hyperphagia, it would be interesting to better understand the origin of this behavior. For instance, their neurobiological response to food (cues) during fasting might provide crucial insight into how these patients, as compared to healthy subjects, manage their behavior while hungry. Direct associations between CNS responsiveness and hormonal and metabolic factors have not yet been studied well. More understanding of the etiology of obesity in PWS is desirable since it can provide guidance for proper prevention and treatment strategies. Therefore, in this study, we aimed to investigate whether adult PWS patients differ from healthy siblings in their response to food stimuli in the fasting state, and if they show differences in the activation of brain areas known to be involved in hunger and food intake. Secondly, we investigated is the existence of an association between the activation of these brain areas and various peripheral factors ((an)orexigenic hormones, metabolites, and IGF-1) in adult PWS patients. By recruiting siblings as healthy controls, we minimalized confounding due to genetic and social factors, with the advantage of reduced between-subject heterogeneity and thus greater experimental power.

## 2. Materials and Methods

### 2.1. Patients

Patients were recruited via the Dutch Prader–Willi patients’ association, as described before [39]. Twelve PWS patients, four males and eight females, with a median age of 22.9 years, participated in this study. All PWS patients had a paternal deletion and were right handed. Patients were excluded if they received GH treatment within three months before enrolment. Five patients were treated with psychotropic drugs: one with risperidone, one with citalopram and risperidone, one with risperidone and valproic acid, and two with modafinil. Written informed consent was obtained from the patients and their parents/caretakers. The study was approved by the Medical Ethics Review Committee of the VU University Medical Center and was conducted according to the principles of the Helsinki declaration.

### 2.2. Healthy Controls (Siblings)

Fourteen healthy brothers and sisters of the PWS patients (seven males and seven females) were included as healthy controls, as described previously [39]. They had a median age of 28.5 years, good general health, and no history of pituitary disease, surgery, or radiotherapy of the head. IGF-1 levels were determined in order to rule out abnormalities of the GH/IGF-1 axis, and IGF-1 levels were normal for age and sex in all healthy controls. Except for one, all controls were right handed. One healthy control was treated with venlafaxine for an earlier depression, which had been in remission for a long time.

### 2.3. Laboratory Tests

Blood samples were drawn between 08:00 and 10:00 a.m. in a fasting state (fasting since 8:00 p.m. of the day before). Standard laboratory tests such as for complete blood count and kidney and liver function were performed in PWS patients to rule out any underlying disease, which revealed no relevant abnormalities. Blood samples for hormonal assessments (Appendix A, Table A1) were drawn at the same time, but were analyzed all together at the end of the study. In healthy controls, only IGF-1 measurements were taken. Both IGF-1 concentration and IGF-1 standard deviation scores (SDS) were used in the analysis. All other hormonal/laboratory assessments were performed at the endocrine laboratory of the VU University Medical Center with commercially available, regularly completed internal and external quality control immunoassays.

### 2.4. Intelligence

Intelligence Quotient (IQ) was measured with the Raven Colored Progressive Matrices (CPM) and the Groningen Intelligence Test (GIT) in both PWS patients and healthy controls, as described in previous studies [44,45,46].

### 2.5. Experimental Tasks

**Figure 1:**
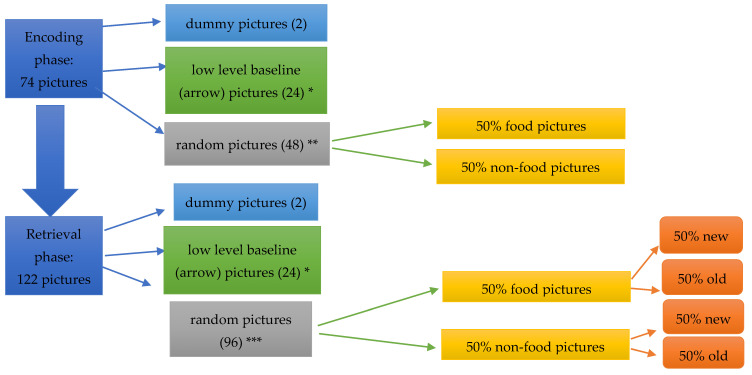
Experimental tasks. * Patients were asked to indicate the direction of the arrow: left of right button. ** Patients were asked to indicate whether pictures were taken indoor (left button) or outdoor (right button). *** Patients were asked to indicate whether pictures were new (left button) or previously seen during the encoding phase (“old”, right button).

MRI scans were obtained at 11:00 a.m. in a fasting state (all participants were fasting since 8.00 p.m. of the day before). First, a T1-weighted structural MRI scan of the brain was performed. Thereafter, functional MRI scans were taken. In this study, echo-planar images with blood oxygenation level-dependent contrast were obtained with a 1.5-T unit (Magnetom Sonata; Siemens Medical Systems, Erlangen, Germany) using a circularly polarized head coil. An echo-planar imaging sequence (TR = 3.310 s, TE = 45 ms, flip angle = 90°) with fat suppression was used to create transversal whole-brain acquisitions (35 slices of 3 mm with 10% gap, voxel size 3.3 mm^3^). As shown in Figure 1, two series of pictures were presented in a block design, depicting food or non-food items such as landscapes, people, and houses (software: E-Prime, Psychology Software Tools Inc., Pittsburgh, PA, USA). These pictures were matched according to visual complexity, both for the objects shown and their background. They were not systematically matched for color [12]. Each picture was displayed for 4000 milliseconds (ms), followed by a pause of 1500–2500 ms, with a preset jitter before the next picture was shown. In the encoding phase, 74 pictures were presented: 2 dummy pictures, 48 randomly selected pictures with food (half) and non-food (half), and 24 low-level baseline pictures (left/right arrow). A button box was used to register the subject’s response and reaction time. In the encoding phase, subjects were requested to press the button to indicate whether the pictures were taken indoor (left button) or outdoor (right button), or to press the button of the direction of the arrow to control for attention differences. Subjects were not asked to memorize the pictures. After the encoding phase, the retrieval phase started in which 122 pictures were presented: 2 dummy pictures, 96 pictures randomly selected with food (half) and non-food (half) (half new, half presented in the encoding phase), and 24 low-level baseline pictures. During the retrieval phase, subjects performed a two-choice recognition task (picture new = left button; picture already seen in encoding phase = right button) to assess memory performance with food versus non-food stimuli.

### 2.6. Statistical Analysis

All baseline characteristics as well as laboratory and psychometric measurements were analyzed using the statistical software package SPSS version 15 (SPSS, Chicago, IL, USA). Since most variables were not normally distributed, all data are presented as median ± IQR (interquartile range); non-parametric tests were used, unless stated otherwise. For differences between the group of PWS patients and the control group, Mann–Whitney tests were performed. Significance level was defined as *p* < 0.05 and all tests were two-tailed.

The Statistical Parametric Mapping (SPM12) software (Wellcome Department of Imaging Neuroscience, Institute of Neurology, London, UK) was used for imaging analysis. fMRI images were realigned, slice timed, and co-registered to the structural MRI scan, then warped to a standard brain template and spatially smoothed using an 8 mm full-width at half-maximum filter. Next, imaging data were analyzed within the context of the General Linear Model, using boxcar regressors convolved with a synthetic hemodynamic response function. For each subject, we performed comparisons of “all pictures (food and non-food)” versus low-level baseline, non-food versus low-level baseline, food versus low-level baseline, and food versus non-food; we then entered the resulting contrast images into second-level (random effects) analyses of variance, as implemented in SPM, using ANOVA. For the present report, contrast images were summed over the encoding and retrieval phases in order to control for attention differences and increase power. Although encoding and retrieval are not identical processes, they are related and their neural substrate shows considerable overlap [47,48]. Moreover, we were primarily interested in visual processing and limbic regions rather than brain regions involved in memory processes per se. Additionally, in the PWS group, analyses of covariance were performed using hormonal parameters as regressors. Results were considered significant if they reached a threshold of *p* = 0.05 corrected for family-wise-error (p_fwe), either at the whole-brain level or within the predefined ROIs (regions of interest), for which a small volume correction was used (centering a 10 millimeter (mm) radius sphere for cortical areas and a 5 mm sphere for subcortical areas around the peak voxels identified in the overall main effects summed over the two groups). Anatomical regions as identified by the Montreal Neurological Institute (MNI) coordinates for peak effects were verified using a standard brain atlas. Based on previous studies, a priori ROIs included anterior insula, amygdala, and fusiform gyrus.

## 3. Results

Baseline characteristics and psychometric measurements of the fMRI task are displayed in Table 1. There were significant differences in PWS patients when compared to healthy controls in height (lower), BMI (higher), IGF-1 concentration and IGF-1 Z-score (lower), and IQ scores on both Raven and GIT (lower).

### 3.1. Psychometric Measurements fMRI Task

Outcomes of reaction time (RT) during the fMRI task for both the encoding and retrieval phases are also shown in Table 1. Only RTs for correct trials were evaluated. In the encoding phase, RTs for baseline, non-food, and food pictures were significantly longer in PWS patients in comparison to controls. However, in the retrieval phase, this was only the case for baseline and old non-food pictures. RTs for new non-food and new food pictures were similar across groups. On the other hand, for old food pictures, RTs were nominally shorter in PWS patients than in controls, although this difference was not statistically significant. With regard to accuracy, as measured by the percentage of correct answers, PWS patients scored lower on both non-food and food items in the encoding phase. In the retrieval phase, accuracy was significantly lower in PWS patients for all pictures, except for new food pictures.

### 3.2. Imaging Data

Across groups, watching food pictures was associated with robust activation in the bilateral dorsal and the ventral visual stream, including the fusiform gyrus and extending into the amygdala, bilateral anterior insula, dorsolateral prefrontal cortex, and the dorsal anterior cingulate cortex (Table 2). A similar pattern was observed for non-food pictures versus baseline. For food pictures versus baseline, analyses of groups according to condition interaction revealed greater activation in controls in the bilateral ventral stream extending into the posterior right parahippocampal gyrus and the bilateral insula, whereas the reverse contrast did not show areas of greater activation in PWS patients. 

For food pictures versus baseline, group by condition interaction analyses revealed greater activation in controls in bilateral ventral stream extending into posterior right parahippocampal gyrus and bilateral insula, whereas the reverse contrast did not show areas of greater activation in PWS patients.

**Figure 2:**
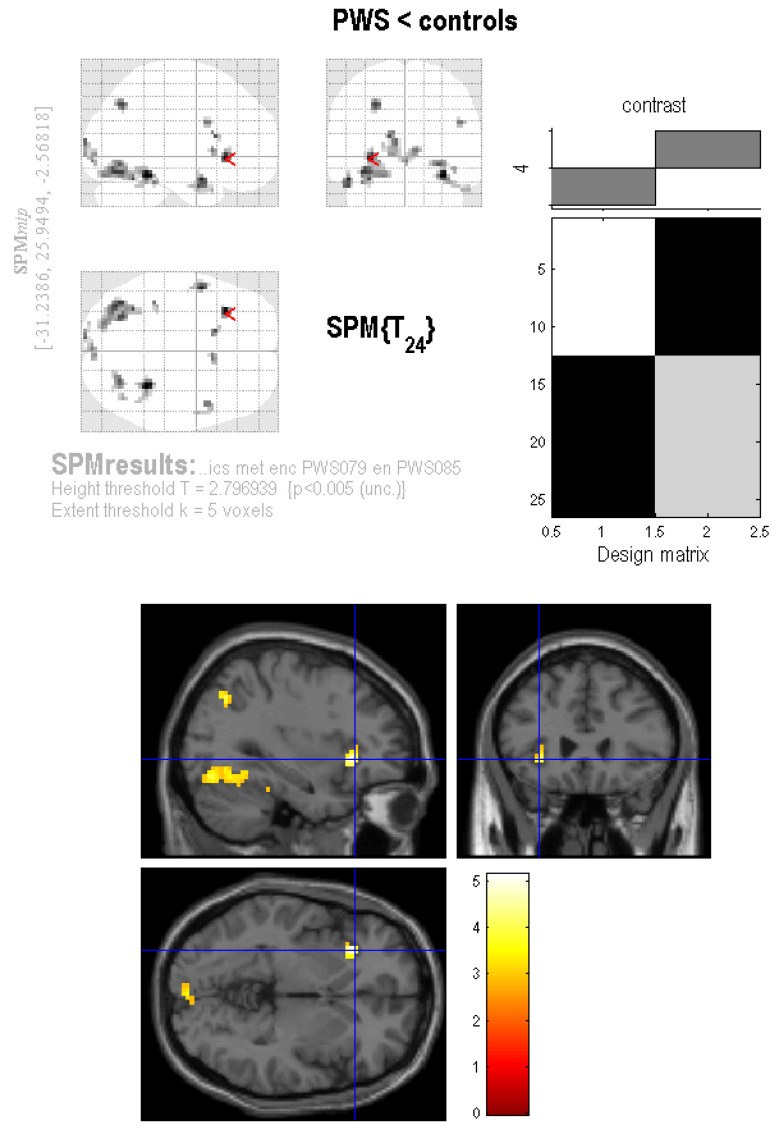
Areas with more brain activation in controls than in PWS patients, for food versus non-food pictures. Upper panel shows area with more brain activation in controls than in PWS patients, for food versus non-food pictures. Lower panel shows significantly higher blood oxygenation level-dependent (BOLD) activation in the left insula in controls than in PWS patients, for food versus non-food pictures (MNI coordinates –33, 24, –3; statistical *Z*-value = 4.06; *p* < 0.004 corrected for family-wise-error).

For non-food pictures versus baseline, controls showed greater activity only in the bilateral ventral visual stream. Again, the reverse contrast did not reveal any significant activation in PWS patients. Finally, the critical comparison of food vs non-food pictures showed significantly greater activation in the left insula (Figure 2) and the bilateral fusiform gyrus in controls, but there were no areas of significantly greater activation in PWS patients (Table 3).

### 3.3. Imaging Data Combined with Endocrine and Metabolic Parameters

Regression analyses of hormonal parameters with fMRI activity in PWS patients watching food versus non-food pictures showed a significant negative correlation with glucose in the right amygdala (24, –3, –21; Z = 3.24; p_fwe = 0.007); for leptin, we observed a positive correlation in the right anterior hippocampus/amygdala (24, –12, –12; Z = 3.59, p_fwe = 0.028). No significant correlations were found for adiponectin, ghrelin, IGF-1, insulin, resistin, or triglycerides.

## 4. Discussion

In the current study, we aimed to investigate whether adult PWS patients differ from healthy siblings in their response to food stimuli in the fasting state and show differences in the activation of brain areas known to be involved in hunger and food intake. Likewise, we investigated whether the activation of these brain areas were associated with various peripheral factors in adult PWS patients. We found that PWS patients reacted slower than healthy controls to the presented baseline and non-food pictures during the fMRI task, while their response time to new or old food pictures in the retrieval phase was similar or even faster, although the latter difference was not significant. Additionally, they had lower accuracy in all conditions, except for new food pictures during the retrieval phase. This suggests that PWS patients are well aware when they are observing new food pictures, and that they have a tendency for faster reactions when looking at food they have seen before. Our imaging data revealed that PWS patients indeed have different food-related brain activation as compared to healthy controls, showing less activation in the left insula and bilateral fusiform gyrus. Moreover, we found that glucose levels were negatively associated with activation in the right amygdala, and leptin levels were positively associated with activation in the right anterior hippocampus and right amygdala in PWS. These findings suggest that within this patient group, aberrant central responses alone as well as their interaction with peripheral satiety signals such as glucose and leptin might play a substantial role in the development of hyperphagia and obesity.

While there is supporting evidence of a delay in the post-meal activation of brain areas known to be involved in satiety in PWS patients, previous neuroimaging data on the central pathways during hunger in these patients are limited and inconclusive [1,19,49]. Some studies on the performance of food-related tasks during fasting show more activation in PWS as compared with controls in the neural regions involved in the anticipation of food during hunger (lateral OFC, inferior temporal cortex) and food-related behavior (hypothalamus and right amygdala). Another study by Holsen et al. (2006) found greater brain responses in controls instead (amygdala, OFC, medial PFC, insula, parahippocampal gyrus, and right fusiform gyrus), which is more in line with our results [20,21,49]. Discrepancies between the mentioned studies and the present study might be due to differences in methodology, i.e., fasting time, age differences (other studies also included children), and comparison to different control groups (healthy versus BMI or cognition-matched). Moreover, the type of presented food might also be essential during the central processing of food stimuli, as was shown by Dimitropoulus and Schultz (2008) who only found differences in PWS patients when looking at high-calorie food [20]. Considering that low and high-calorie foods were not analyzed separately in the present study, this might also have affected the results. Lastly, in an fMRI study by Jakobsdottir et al. on fifteen healthy men who performed the same experimental tasks within the same fasting period as in our study, food-related neural activation appeared to be comparable to that of our control group, including the ventral visual stream, bilateral fusiform gyrus, and the hippocampus [11]. In the present study, such responses were not observed in patients with PWS, indicating that central food processing during hunger is different in PWS. No associations for peripheral factors (ghrelin, insulin, leptin, glucose, and free fatty acids) were found and the authors recommended more research on adiponectin and glucagon-like peptide 1 (GLP-1) as possible modulators. The present study included adiponectin, but did not find a correlation in PWS patients.

We found that controls showed greater responses in the insular region and the fusiform gyrus as compared to PWS. The insula is known to participate in many functions, including receiving visceral and olfactogustatory sensations, emotional decision-making, and salience [50]. Case studies have shown that lesions in this area can, amongst others, lead to decision-making deficits under risk and affect taste [51,52]. Interestingly, previous research in PWS concerning taste showed a positive correlation between self-reported ratings of disgust after watching a movie containing disgust-provoking food scenes, such as maggots and worms, and responses in the insular region, while these individuals showed less problems eating contaminated food and strange (in)edible food combinations as compared to (IQ-matched) controls [53,54]. Since PWS patients are also known to exhibit pica behavior, it would be interesting to gain more insight into food-related behavior and insular function in PWS individuals. The fusiform gyrus is part of the inferior temporal and occipital cortex, and is historically known to play a key role in face and object recognition. However, more recent studies also suggest that it is involved in hunger and reward processing [13,55,56]. Since PWS patients show high food motivation behavior, we expected to find more activation in this area in PWS individuals than in controls when looking at food pictures during fasting. However, our results show the opposite. It could also be argued that PWS patients might experience less feelings of reward, thereby stimulating the continuation of eating and seeking food. The negative correlation we found between glucose levels and activity in the amygdala may support this explanation since the amygdala plays a crucial role in the central motivation/reward circuitry; thus, the lower the glucose (i.e., during fasting), the higher the motivation for and rewards out of eating. In our study, we observed a positive correlation between leptin concentrations and brain activation in the amygdala region. Although leptin is seen as an anorexigenic hormone, increased levels of leptin in obesity have previously been associated with more activity in the motivation/reward circuitry while looking at food pictures [57]. This indicates that in PWS, alterations of leptin levels and central processing might lead to enhanced food-related reward sensations, thereby stimulating hyperphagia and obesity. Altogether, we found that in PWS adults during hunger, food-related brain activity in regions related to taste, decision-making, and reward processing may be altered, and that leptin might be a contributing factor to hyperphagia in PWS individuals.

Although GH and IGF-1 receptors are distributed over the entire CNS, including the (hypo)thalamus, amygdala, and hippocampus, no correlation between IGF-1 and food-related neural activity during fasting in PWS individuals was found. This implies that the positive effect of GH replacement therapy on body composition in adult PWS patients is more likely attributable to metabolic than neural effects of IGF-1 [58].

In order to study food-related brain activation, we used task-evoked blood-oxygen-level-dependent (BOLD) signal changes to reflect neuronal activity, which is based on the principle that an increase in blood flow occurs where more neurons are activated. We assumed that the patients were well able to perform the psychometric tasks, thereby leading to the neural activation of interest. One of the problems of the BOLD technique lies in the fact that the vasculature of the brain is highly heterogeneous and signal changes in brain regions with low capillary density, such as parts of the entorhinal cortex and hippocampus, are more difficult to detect [59].

There is still little known about direct associations between CNS responsiveness (functional imaging data) and neuroendocrine and metabolic factors during hunger in PWS. One of the methodological strengths of the current study is the inclusion of a healthy control group consisting of siblings, thereby ruling out genetic and social factors as much as possible. Furthermore, PWS patient groups in previous studies often consisted of both children and adults, while our study only included adult patients. The current study also has its limitations. PWS patients had lower intelligence scores, which might have affected psychometric results, considering that intelligence is correlated with mental speed and reaction time [60]. Nevertheless, that does not explain the differences found in reaction time to food versus non-food pictures since one would expect that all reaction times would be longer. The use of psychotropic medication, which was more prevalent in the PWS group, could have influenced reaction times, body composition, and/or brain activation. However, in our belief, stopping these medications could cause potential danger and was not ethically justified. Unfortunately, we were not able to make comparisons between the genetic subtypes of PWS individuals since all patients had the paternal deletion variant. Although the same prevalence of obesity is found in the most common genetic variants, it would be interesting to compare central food regulation and hormonal factors between the different genetic subtypes of PWS [61].

Nowadays, PWS patients are getting older and curative treatments are lacking. To date, most of the interventions meant to achieve long-term weight reduction have failed, making the prevention of obesity and its complications extremely important, although this often remains a challenge. The development of appropriate preventive measures and (non)pharmacological treatment strategies should focus on factors contributing to hyperphagia and obesity. By performing this study, we contributed to unravelling the underlying central pathways involved in hunger and the relationship between brain regions and several peripheral modulating factors in adult PWS patients. For the future, it would be interesting to perform an fMRI study in which the relationship between hyperphagia, GLP-1, and neural regions involved in hunger and satiety are assessed in PWS patients, since GLP-1 agonists are known to lower feelings of hunger and increase feelings of fullness, and are therefore currently used in the treatment of obesity [62]. Furthermore, future studies could focus on the role of insular involvement in the development of obesity in the different genetic subtypes of PWS.

## 5. Conclusions

Altogether, we conclude that PWS individuals show aberrant food-related brain activation during fasting, with less activation in regions involved in olfactogustatory sensations, emotional decision-making, and reward processing when compared to their healthy siblings. In addition, leptin was associated with responses in the neural motivation/reward circuitry, thereby possibly contributing to hyperphagia. More research is needed on the integration of the current results into proper obesity prevention and management strategies within this patient group.

## Figures and Tables

**Table 1:** Baseline characteristics and psychometric measurements of the fMRI task.

	PWS						Controls					
	Total Group (*n* = 12)		Men (*n* = 4)		Women (*n* = 8)		Total Group (*n* = 14)		Men (*n* = 7)		Women (*n* = 7)	
	Mdn	IQR	Mdn	IQR	Mdn	IQR	Mdn	IQR	Mdn	IQR	Mdn	IQR
Age (years)	22.9	14.5	26.6	18.3	22.3	14.6	28.5	18.0	28.4	17.4	28.7	20.1
Height (m)	1.57 *	0.11	1.60	0.09	1.54	0.10	1.77	0.17	1.86	0.07	1.69	0.05
Weight (kg)	69.9	36.1	67.9	32.9	77.7	39.5	73.1	15,9	79.1	15.9	65.2	14.1
BMI (kg/m²)	29.8 *	17.4	26.1	13.2	34.3	19.1	22.4	4.6	22.7	5.1	22.0	5.8
IGF-1 (nmol/L)	15.4 **	7.3	16.0	11.9	15.4	9.5	21.8	11.6	21.4	14.2	22.1	10.5
IGF-1 Z-score (SDS)	−1.9 **	1.1	−1.5	1.24	−1.95	0.90	−0.79	1.1	−1.00	1.1	−0.76	1.7
Adiponectin (mg/L)	14.2	6.3	13.2	9.1	14.2	5.1						
Ghrelin (ng/L)	2118	2381	2118	2201	2151	2425						
Glucose (mmol/L)	4.1	0.6	4.2	0.2	4.0	1.1						
Insulin (pmol/L)	31.0	31.2	37.2	179.1	28.2	31.6						
Leptin (µg/L)	23.2	30.7	20.1	38.7	29.0	30.7						
Resistin (ng/mL)	5.1	3.8	4.3	5.7	5.1	3.7						
Triglycerides (mmol/L)	0.80	0.3	1.75	2.6	0.80	0.4						
IQ GIT	41 **	12	40	3	43	18	87	35	93	48	85	20
IQ RAVEN	65 **	10	70	15	65	10	110	17	110	16	119	25
% correct encoding baseline	100	24					100	0				
% correct encoding non-food	76 **	45					96	5				
% correct encoding food	61 **	25					79	16				
RT correct encoding baseline (ms)	1209 **	654					680	209				
RT correct encoding non-food (ms)	1676 **	489					1075	399				
RT correct encoding food (ms)	1747 **	615					1233	658				
% correct retrieval baseline	96 **	29					100	0				
% correct retrieval non-food new	89 *	62					96	4				
% correct retrieval non-food old	63 **	26					83	14				
% correct retrieval food new	86	46					94	6				
% correct retrieval food old	39 **	41					73	35				
RT correct retrieval baseline (ms)	1065 **	651					718	148				
RT correct retrieval non-food new (ms)	1260	317					1238	444				
RT correct retrieval non-food old (ms)	1676 *	892					1353	333				
RT correct retrieval food new (ms)	1347	327					1307	383				
RT correct retrieval food old (ms)	1476	1225					1621	454				

All data are presented as median (IQR). * Significant difference when compared to control group, *p* < 0.05 (** *p* < 0.01). Mdn: Median; IQR: Interquartile Range; BMI: Body Mass Index; IGF-1; Insulin-Like Growth Factor 1; SDS: Standard Deviation Score; IQ: Intelligence Quotient; GIT: Groningen Intelligence Test; RT: reaction time.

**Table 2:** Main effect of food pictures versus baseline across groups.

ROI	MNI-Coordinates			k	*Z*-Value	*p*-Value
	x	y	z			
R occipital cortex	39	–81	9	5367	7.19	<0.0001
L occipital cortex	–39	–81	–12	5367	6.55	<0.0001
R fusiform cortex	33	–45	–12	5367	7.32	<0.0001
L fusiform cortex	–33	–51	–15	5367	6.63	<0.0001
R dorsomedial prefrontal cortex	6	21	48	385	5.31	0.003
R insula	36	24	0	443	5.07	0.009
R dorsolateral prefrontal cortex	45	6	33	443	5.13	0.007
L dorsolateral prefrontal cortex	–51	9	30	298	5.34	0.001
L insula	–30	24	3	199	5.29	0.003

ROI: region of interest; MNI: Montreal Neurological Institute; R: right; L: left; k: voxel extent.

**Table 3:** Interaction effects of watching food versus non-food pictures.

	Controls > PWS Patients					
ROI	MNI-Coordinates			k	*Z*-Value	*p*-Value
	x	y	z			
R fusiform gyrus	33	–45	–18	13	4.18	0.003
L fusiform gyrus	–33	–57	–21	36	3.69	0.013
L insula	–33	24	–3	20	4.06	0.004
	**PWS patients > controls**					
	No significant voxels					

ROI: region of interest; MNI: Montreal Neurological Institute; R: right; L: left; k: voxel extent.

## Data Availability

The data presented in this study are available on request from the corresponding author. The data are not publicly available due to privacy restrictions.

## Appendix A

**Table A1:** Hormonal assessments.

Laboratory Parameter	Lab Method	Company	Lower Limit of Quantitation	Mean intra-Assay % CV	Reference Values
IGF-1 and IGFBP-3	Immunometric assay, Luminescence	Immulite 2500 ^®^ laboratory assay, Siemens Medical Solutions Diagnostics, USA			sex- and age-specific
Insulin	Immunometric assay, Luminescence	Advia Centaur, Siemens Medical Solutions Diagnostics, USA	10 pmol/L	20 pmol/L: 4% 500 pmol/L: 3% 500 pmol/L: 4%	12–96 pmol/L *
Adiponectin	Radioimmunoassay	Linco Research Inc., St. Charles, MO, USA	0.5 mg/L	whole range: 5%	0.8–48 mg/L
Ghrelin	Radioimmunoassay	Linco Research Inc., St. Charles, MO, USA	240 ng/L	whole range: 4%	800–3000 ng/L
Leptin	Radioimmunoassay	Linco Research Inc., St. Charles, MO, USA	0.5 μg/L	5 μg/L: 8%25 μg/L: 3%	Men **: 2.5–8 μg/L Women **: 4.5–16 μg/L
Resistin	Immnometric assay (colorimetric)	BioVendor Laboratory Medicine, INC, Modrice, Czech Republic	0.8 ng/ml	whole range: 5%	4.1–12.1 ng/ml

IGF-1: Insulin-Like Growth Factor 1; IGFBP-3: Insulin-Like Growth Factor Binding Protein 3; CV: Coefficient of variation;* Fasting. ** BMI 18–25 kg/m².
